# Application of the Delphi methodology to forecast competencies oriented towards AI-driven disinformation detection in an expanded DigComp framework

**DOI:** 10.1016/j.mex.2025.103401

**Published:** 2025-06-01

**Authors:** Cristina M. Arribas, Rubén Arcos, Manuel Gertrudix

**Affiliations:** Department of Audiovisual Communication and Advertising, University Rey Juan Carlos (ROR 01v5cv687), Camino del Molino 5, Fuenlabrada, Madrid 28942, Spain

**Keywords:** Artificial Intelligence, Disinformation, Foreign information manipulation and interference, Digital literacy, Delphi study, DigComp, Delphi Method

## Abstract

The growing development of capabilities, techniques, and technologies associated with Artificial Intelligence opens a new scenario full of opportunities for Foreign Information Manipulation and Interference, which presents an added challenge for detecting and countering disinformation content. Within the preventive strategies to combat these threats, digital literacy emerges as essential. The aim of the research, whose method is detailed here, is to proactively identify emerging technological trends that will shape the disinformation and FIMI ecosystem over the next 10 years, and to propose competency-based training and curricular models to address these challenges in their latent phase.

• The article introduces a Delphi study model designed to identify training needs to effectively combat disinformation and FIMI in the context of generative AI and other emerging technologies.
• A detailed guide of the conducted study is provided, intended to be replicated in future research or to support the development of subsequent studies aimed at understanding the training needs arising from new technologies applied to misinformation.
• Questionnaires and datasets are provided with results that enable comparative, longitudinal, and replication studies

## Graphical abstract

**Figure:**
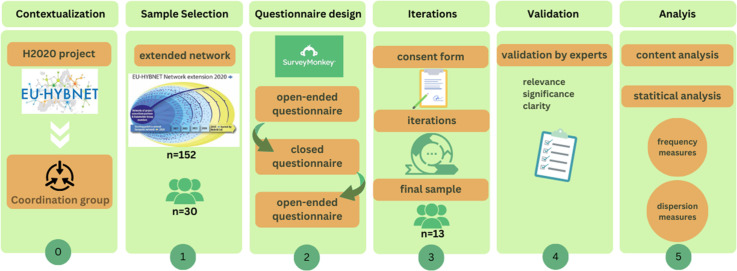
Diagram with the phases of the methodology.

Specifications table

**Table:** 

Subject area:	Computer Science
More specific subject area:	Generative AI, communication and education
Name of your method:	Please write the name of the method you are describing in this article
Name and reference of original method:	C.M. Arribas, R. Arcos, M. Gertrudix, Rethinking education and training to counter AI-enhanced disinformation and information manipulation in Europe. A Delphi Study. Datasets, 2024a, https://doi.org/10.5281/zenodo.15212421 C.M. Arribas, R. Arcos, M. Gertrudix, Rethinking education and training to counter AI-enhanced disinformation and information manipulation in Europe. A Delphi Study. Initial questionnaire (Q1), 2024b https://doi.org/10.5281/zenodo.11852909 C.M. Arribas, R. Arcos, M. Gertrudix, Rethinking education and training to counter AI-enhanced disinformation and information manipulation in Europe. A Delphi Study. Second questionnaire (Q2), 2024c, https://doi.org/10.5281/zenodo.11851800
Resource availability:	Surveymonkey: https://www.surveymonkey.com/

## Background

Artificial intelligence, along with other new and emerging technologies and their potential use to create and disseminate disinformation, poses an additional challenge to what has become one of the greatest threats of our time. Among the existing approaches, the development of detection models [[1], [2], [3]] for this type of content requires continuous adaptation to a rapidly changing ecosystem, leading to a cat-and-mouse game dynamic [4]. On the other hand, strategies based on monitoring emerging trends through technology monitoring systems and early warning systems to identify latent problems [5], as well as thematic trends and new disinformation tactics, are an essential complementary approach to act proactively.

Likewise, within preventive approaches, the importance of identifying training needs and the skills required to combat AI-driven disinformation is emphasized, with the aim of incorporating them into citizens' educational curricula. This article details the methodology used in a Delphi study designed for the early identification of competencies aimed at detecting AI-assisted disinformation, as well as other emerging technologies and trends associated with tactics that may pose a challenge for mitigating the phenomenon of disinformation and Foreign Information Manipulation and Interference (FIMI).

The relevance of the Delphi study is justified by the emergency of the addressed issue and the attention that digital competency has received as a preventive strategy in the fight against disinformation and FIMI.

The relevance of the study is justified by the urgency of the addressed issue [6,7] and the importance of digital competency as part of preventive strategies in the fight against disinformation, which prioritizes the need to develop and update specific competencies and skills in response to new and emerging technologies, especially generative AI.

This study has been developed inside the EU-Hybnet project (Empowering a Pan European Network to counter hybrid threats) funded by the European Union's Horizon 2020 research and innovation program under agreement No883054. The project aims to strengthen European networks combating hybrid threats through the proliferation of knowledge and cooperation between industry, professionals, and academia, as well as through the development of advanced solutions for network collaboration, ensuring their long-term sustainability [8]. The research addresses the project's objective 6: 'to foster capacity building and knowledge exchange to counter hybrid threats.

The study takes as a reference the concept of digital competence outlined in the DIGCOMP report: A Framework for Developing and Understanding Digital Competence in Europe [9] by the European Commission, later developed in the first version of the Digital Competence Framework for Citizens (2013). In this, digital competence is defined as “the confident, critical and responsible use of, and engagement with, digital technologies for learning, at work, and for participation in society. […] a combination of knowledge, skills and attitudes.” [10]. Additionally, the study takes as a starting point for identifying the formative gaps the five areas of competence delineated in DigComp version 2.2 [11]: Information and data literacy, Communication and Collaboration, Digital Content Creation, Safety, and Problem Solving.

### Method details

**Fig. 1:**
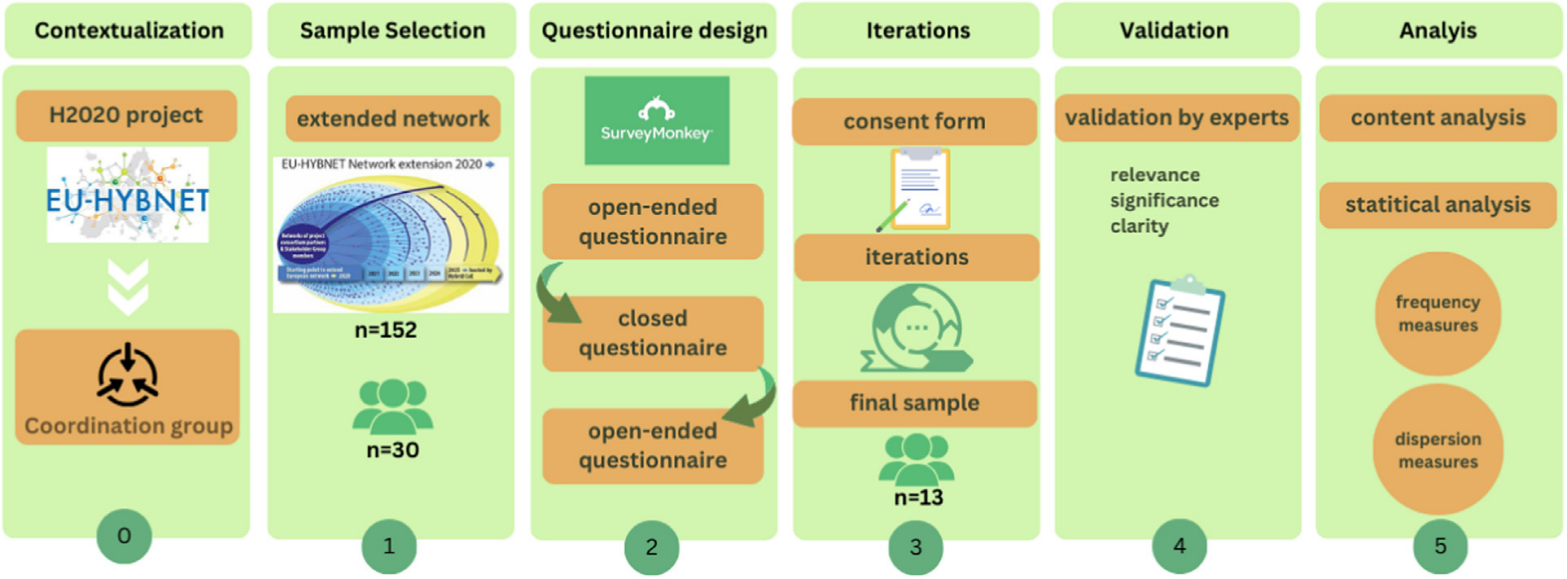
Succinctly illustrates the phases in which the Delphi study is developed, as explained throughout this article.

The study aims to identify emerging training needs to define a framework of specific competencies and skills to effectively combat disinformation/FIMI in the face of new and developing technologies (Fig. 1).

### Phase 0. Contextualization

The research was proposed within the framework of the third cycle of the EU-HYBNET project. The analysis methods were defined during a coordination meeting, included the study protocol, the selection of experts, the schedule, the data collection systems, the validation and consensus assessment procedure and a preliminary guide with the items for the first round if iterations. Additionally, a coordination group to support the process was created.

The Delphi method was selected by its valuable predictive power for obtaining expert consensus in contexts of uncertainty or where information possibilities are scarce, facilitating the systematic exploration and forecasting of emerging issues. The method was created in the 1940s by researchers at the Rand Corporation to predict the impact of technology on warfare [12]. Today, it is applied to a wide range of topics, gaining prominence in areas such as digital competencies and technological forecasting [13,14], highlighting its relevance for addressing the challenges of AI and the developing competences in higher education [15].

### Phase 1. Sample selection

The selection of the panelists was based on the criterion of belonging to the extended network of the EU-HYBNET project, which consists of more than 120 organizations, including academia, SMEs (small and medium-sized enterprises), and public and private organizations. Additionally, 5 contacts of the members of the coordination group were added. This ensured a high level of expert knowledge in the study's subject matter. The selection process focused on experts in hybrid threats, digital transformation, and AI-driven disinformation to ensure alignment with the study's goals.

The initial sample included 30 experts who were sent a formal invitation via email with the study details and informed consent. This sample size aligns with previous methodological recommendations. Delbecq, Van de Ven, and Gustafson [16] suggest involving 10–15 participants as ideal, with a minimum of 7 and no more than 30. Needham and Loë [17] recommend between 10 and 30 participants if they come from the same discipline. De Villiers, De Villiers, and Kent [18] advise a panel of 5 to 10 experts when the experts come from different disciplines. Powell [19], on the other hand, suggests that representation should be evaluated based on the expertise of the panel members rather than their number. The final panel comprised security experts, stakeholders, academics, and representatives from SMEs, thereby encompassing the various types of experts identified by Landeta [20]. 57 % of the participants worked in academia and 43 % were practitioners. All of them were senior managers, with 78 % holding a PhD. They represented 12 different European nationalities, and two were from the United States. The response rate was 46.67 %. One of the panellist did not complete the study, so the final response rate was 43.33 %.

### Phases 2 and 3. Design of questionnaires and iterations

The first online questionnaire (Q1) used a SurveyMonkey panel (2023). This tool was selected for its versatility, given the complexity of the instrument designed for data collection. Its customization features, advanced logic for creating personalized survey paths, and integration with other tools and platforms enable more efficient data collection and analysis.

The questionnaire (Q1) included 5 open-ended exploratory questions to obtain the initial perspectives of the experts. This questionnaire model is common in most Delphi studies as a starting point for subsequent rounds of closed and specific questions [21]. The first questionnaire aimed to address the three specific objectives set out and the questions related to them (Table 1). The questions aimed to identify gaps and weaknesses in current competencies related to disinformation and FIMI, develop a ten-year prospective landscape assisted by new and emerging technologies, and determine the competencies and curricular models that are most appropriate for the different groups of students. Some trends or consolidated issues discussed during the coordination meeting were suggested to the participants into the questions to help focus the content of their responses. Additionally, a curricular brief was required to the participants. The data was subsequently processed to extract statistical information about the level of experience, academic background, professional field and area of specialization.

**Table 1:** First questionnaire structure and relation between questions, research questions and objectives.

Objectives	Research question	Questions
O1. Identify emerging training needs to counter disinformation in a context of polarisation, the rise of political populism and the development of new forms of digital authoritarianism. O2. Define the framework of specific competences to combat AI-based disinformation and information manipulation. O3. Describes the units of competency that need to be developed to address the education/training gaps related to the impact of new technologies such as synthetic content, immersive communications, virtual reality and others in the fight against hybrid threats and cognitive warfare.	R1. What challenges and trends in the next ten years will shape the disinformation and FIMI landscape? R2. What is the current knowledge and what are the competencies required to address disinformation and FIMI? R3. What competencies and skills will be needed to tackle disinformation and FIMI in the next ten years? R4. How will training models and educational curricula impact training programs and formal education?	Q1. In ten years’ time, what do you think will be the most important issues or threats that need to be addressed regarding disinformation and FIMI? Q2. How would you describe the current level of knowledge and skills available to deal with modern audio-visual manipulation and generative AI-based disinformation and FIMI (foreign information manipulation and interference)? Q3. In this time frame, what do you think are the biggest challenges for education and training to adequately combat these threats? What knowledge and skills do you think will be necessary for both practitioners and ordinary citizens? Q4. What specific competences will be needed in the next ten years to successfully address and counter disinformation and information manipulations in a context of advanced technologies such as artificial intelligence, immersive environments based on mixed, virtual, and augmented reality and others? Q5. What impact will this have on formal education and training, considering the 10-year period?

An email with the instructions and the aim of the study, along with an informed consent form, was sent to the selected panellists. 20 agreed to participate and 14 completed the first round. The completion of this round took 5 weeks.

The second round (Q2) was developed based on the analyzed results obtained in round 1. During this round, the DigComp framework was included to guide the participants and obtain more specific and actionable responses. The questionnaire was developed in three different blocks. A first block aimed to identify the level of agreement on the responses from round 1(Q1) related to the implications of new and emerging technologies and other trends in Disinformation and FIMI. Panellists were also asked about their level of agreement on the current DigComp's degree of updating, the level of implication of specific emerging and new technologies to tackle disinformation and FIMI, and their perspectives on suitable curricular models in the long term. This block included 9 questions rated on an 11-point Likert scale (0: strongly disagree - 10: strongly agree) and a confidence scale (0 % low confidence - 100 % high confidence). Additionally, a 6-point Likert scale (0: strongly disagree - 5: strongly agree) was included to assess the level of agreement on specific DigComp competency areas for the two target publics (average citizens and practitioners) (Table 2).

**Table 2:** Second questionnaire.

Ítem	Content
1.There is a generally low level of understanding (the public and policymakers) on the phenomenon and of the necessary competences for dealing with audio-visual manipulation and generative AI-based disinformation and FIMI.	Please indicate on a scale of 0-10 your degree of agreement. Please indicate your degree of confidence in your judgment (0-100 %).
2. Some respondents suggest that, even if in the case of practitioners, the current degree of competence is mostly sufficient, it is also true that the fast pace at which technology is evolving, presents a challenge for practitioners to adapt their skills. In particular those about: the analysis of manipulations and conduction of forensics; generative AI; conversational AI; Augmented Reality; and neuro-technology.	Please indicate on a scale of 0-10 your degree of agreement. Please indicate your degree of confidence in your judgment (0-100 %).
3. In ten years, advanced forms of disinformation/FIMI through the use of generative AI and other technologies will be widespread and dominant and will require a proficient level of competence by practitioners.	Please indicate on a scale of 0-10 your degree of agreement. Please indicate your degree of confidence in your judgment (0–100 %).
4. The current DigiComp Framework should be expanded with additional areas of competence aimed at practitioners, for providing contextual knowledge on disinformation/FIMI (e.g. threat actors, geopolitical conflicts, historical revisionism) and other additional skills (e.g. detection and analytic techniques, argument-checking).	Please indicate on a scale of 0–10 your degree of agreement. Please indicate your degree of confidence in your judgment (0–100 %).
5. Career-long continuous training through advanced specialized courses (micro-credentials) on new digital and cyber technologies should be adopted as a strategy by organizations of practitioners to keep updated to deal with disinformation/FIMI in the next ten years.	Please indicate on a scale of 0–10 your degree of agreement. Please indicate your degree of confidence in your judgment (0–100 %).
6. Regarding citizens/average users, the curricula in higher education institutions should be adapted to provide and strengthen those digital competences of the DigComp Framework to all graduates in order to address advanced and AI-enhanced disinformation/FIMI	Please indicate on a scale of 0–10 your degree of agreement. Please indicate your degree of confidence in your judgment (0–100 %).
7. A separate strategy and curricula targeting vulnerable populations that are unlikely to receive education and competences in the DigiComp framework (through higher education institutions) should be developed and adopted.	Please indicate on a scale of 0–10 your degree of agreement. Please indicate your degree of confidence in your judgment (0–100 %).
8. The existing DigiComp framework does not include competences such as radicalization awareness, political systems literacy, scientific attitude, or formal logic and reasoning processes. These are competences deemed to be necessary for dealing with disinformation/FIMI in the next ten years.	Please indicate on a scale of 0–10 your degree of agreement. Please indicate your degree of confidence in your judgment (0 -100 %).
9. Please indicate on a scale of 0–10 your assessment of the relative degree of impact and application of the technologies below in disinformation/FIMI campaigns and your degree of confidence in your judgment (0–100 %) in the ten years´s time.	Please indicate on a scale of 0–10 your degree of agreement. Please indicate your degree of confidence in your judgment (0–100 %).
9.1. Generative Al	Please indicate on a scale of 0–10 your degree of agreement. Please indicate your degree of confidence in your judgment (0–100 %).
9.2. Virtual Reality (VR) and Augmented Reality (AR)	Please indicate on a scale of 0–10 your degree of agreement. Please indicate your degree of confidence in your judgment (0–100 %).
9.3. Neurotechnology	Please indicate on a scale of 0–10 your degree of agreement. Please indicate your degree of confidence in your judgment (0–100 %).
9.4. Quantum Computing	Please indicate on a scale of 0–10 your degree of agreement. Please indicate your degree of confidence in your judgment (0–100 %).
9.5. Internet of Things (loT)	Please indicate on a scale of 0–10 your degree of agreement. Please indicate your degree of confidence in your judgment (0–100 %).
9.6. Next Generation Networks	Please indicate on a scale of 0–10 your degree of agreement. Please indicate your degree of confidence in your judgment (0–100 %).
10. Considering the European Commission´s DigComp Framework below, please rate on a scale of 0 to 5 your estimate on the current level of competence for practitioners regarding the following aspects:	
10.1. Information and data literacy	Please indicate on a scale of 0–5
10.2 Communication and collaboration	Please indicate on a scale of 0–5
10.3. Digital content creation	Please indicate on a scale of 0–5
10.4. Safety	Please indicate on a scale of 0–5
10.5. Problem solving	Please indicate on a scale of 0–5
11 Considering the European Commission’s DigComp Framework below, please rate on a scale of 0 to 5 your estimate on the current level of competence for b) citizens, regarding the following aspects:	Please indicate on a scale of 0–5
11.1. Information and data literacy	Please indicate on a scale of 0–5
11.2. Communication and collaboration	Please indicate on a scale of 0–5
11.3. Digital content creation	Please indicate on a scale of 0–5
11.4. Safety	Please indicate on a scale of 0–5
11.5. Problem solving	Please indicate on a scale of 0–5
12. Please, indicate below and with the more degree of detail as possible (specific areas of competence other than the 5 in the DigComp Framework and skills), competences that you considered are missed and hence should be added to the current DigComp Framework in the training curriculum of practitioners (e.g. digital content forensics) for successfully dealing with current state of the art disinformation/FIMI and AI-enhanced information manipulations.	
12.1. Information and data literacy	Open question
12.2. Communication and collaboration	Open question
12.3. Digital content creation	Open question
12.4. Safety	Open question
12.5. Problem solving	Open question
12.6. Other areas of competencies that you think are necessary.	Open question
13. Please, indicate below and with the more degree of detail as possible (specific areas of competence other than the 5 in the DigComp Framework and skills), competences that you considered are missed and hence should be added to the current DigComp Framework in the training curriculum of average users (e.g. deepfake detection) for successfully dealing with current state of the art disinformation/FIMI and AI-enhanced information manipulations.	
13.1. Information and data literacy	Open question
13.2. Communication and collaboration	Open question
13.3. Digital content creation	Open question
13.4. Safety	Open question
13.5. Problem solving	Open question
13.6. Other areas of competencies that you think are necessary.	Open question

Finally, the questionnaire was supplemented with an open-ended question to allow experts to develop competencies and skills that, according to their judgment, are lost in the five areas of the DigComp 2.2 [22] to successfully dealing with modern disinformation/FIMI and AI-supported information manipulation. This tool was designed to point out specific gaps for both practitioners and citizens The purpose of the multi-question assessment regarding DigComp´s competences was to identify the perceived strengths and weaknesses of the competences rather than to paint an accurate and representative picture of their status. The questionnaires are available in Arribas et al. [23,24].

The use of different Likert scales was related to the level of detail required for each item. For the questions aimed at evaluating the DigComp areas, a smaller scale was chosen, as the level of detail was later explored in the open-ended questions.

Additionally, the agreement scale was complemented with a trust scale in order to achieve a better understanding of participants' attitudes and trust levels through cross-validation of the results. Thus, if the results of both scales are consistent with each other, the confidence in the validity of the collected data is greater. The inclusion of the trust scale is based on experiences from previous Delphi studies [25] que apuntan a la falta de seguridad de algunos expertos al tratar temas complejos y cómo las escalas de confianza son utilizadas para ponderar las respuestas. For this study it was useful since not all experts had the same level of knowledge about the technologies or came from different fields of specialization.

The content of the questionnaires underwent a validation process by experts [26]. For the validation of analysis instruments, there is no consensus on the number of experts that should participate. Authors like Almarasreh [27] suggest a number of 5 to 7 experts to consider the evaluation valid. However, other authors prioritize the level of experience and knowledge of the validators over the quantity, emphasizing the importance of the validators' experience and knowledge [28].

### Phase 4. Validation

In this study the validation process was carried out by 3 members of the project's extended network, who are recognized experts in the field of study. No distortion or biases in the results were considered due to the breadth of the network (more than 120 organizations). The validation process focused on measuring the relevance, significance, and clarity of the questions [29], as well as the proposed measurement scales and their suitability to the different items. Adjustments were made to two of the questions in response to the evaluation.

After validation, the questionnaire was sent to the panelists. It took several reminders to complete the task. Finally, one of the experts left the round. Thirteen completed the study. The completion of this round took 15 weeks. In the Delphi method there is no homogeneously extended opinion on how to establish consensus. This partly occurs because there is no a common definition of the concept, and the confusion with other concepts, such as stability (the consistency of data across different rounds), must be considered [30].

Different thresholds were agreed upon to determine consensus. Consensus was set at 1 for 5-point Likert scales [31,32] and at 2 for 10-point scales [33]. For the coefficient of variation, an optimal consensus threshold of 0.5 was established [34] and acceptable, depending on the context, a result less than 0.8 [35]. For descriptive measures, the consensus threshold of 60–80 % defended by Rowe and Wright (1999) [36] or 75 % by Barrios et al. [37] was used as a reference.

### Phase 5. Analysis

The responses from the first questionnaire (Q1) were qualitatively analyzed by organizing, categorizing, and identifying common themes. Word counts and frequency measures were applied. The technologies mentioned by the experts were grouped thematically in order to recognise emerging patterns. Particular attention was paid to topics that were rarely mentioned but have a high potential for future disruption. It concluded that it was necessary to delve deeper into the scope of competencies. Therefore, it was decided to incorporate the DigComp 2.2 framework in the second questionnaire to better identify training needs.

The content of the responses for the second questionnaire (Q2) was analyzed using both descriptive and inferential statistical methods. Data processing was conducted using IBM SPSS Statistics (v30) and Python within the Google Colab enviroment. In the Python-based analyses, key libraries included pandas and scipy.stats for statistical computations, as well as matplotlib and seaborn for data visualisation.

The multi-question assessment results regarding the level of competence for the five DigComp´s areas were classified into three levels: high, medium, and low, using a 0 to 5 scale. This categorization approach follows standard competence assessment models, such as the European e-Competence Framework (e-CF) (CEN, 2018) [38], which also employs a five-point scale for evaluating competences. The results on the level of agreement for the remaining questions (0 to 10) were grouped into five ranges: high agreement (10–9), agreement (8–7), somewhat agreement (6–5), somewhat disagreement (4–2), and disagreement (2–0). The results for the confidence scale were grouped into four ranges: very high confidence (100–75 %), high confidence (74–50 %), moderate confidence (49–25 %), low confidence (24–0 %). To ensure comparability across scales with different formats, all results were normalized using z-scores.

The analysis included descriptive measures such as frequency, measures of central tendency (mode and mean), and measures of dispersion such as standard deviation, coefficient of variation, and interquartile range [39]. These indicators made it possible to identify elements with high variability or strong consensus and helped to assess the stability and consistency of expert opinions. The datasets can be found in Arribas et al. [40].

The results showed high consensus scores on items 1, 2, 3, 5, 6, 7, and 8, both on the agreement scale (10 points) and the confidence scale. A detailed summary of these results, including coefficient of variation (CV) values for both agreement and confidence metrics, is provided in Table 3. The consensus for the items 9.2, 9.4 and 9.6 results more moderate according to frequency measures, however, they were acceptable (below the threshold of 0.5) according to the coefficient of variation. These results are explained by the lack of familiarity of experts with new or emerging technologies whose effects or implications for disinformation or FIMI are not yet explored.

**Table 3:** Validation criteria.

Ítem	Content
1. Relavance	
1.1 Is the question relevant for evaluating AI-driven disinformation/FIMI detection and other technologies?	-Completely agree
	-Agree
	-Neither agree nor disagree
	-Disagree
	-Completely disagree
1.2. Does the question address important and current aspects of disinformation detection?	-Completely agree
	-Agree
	-Neither agree nor disagree
	-Disagree
	-Completely disagree
1.3 Does the question adequately reflect real-world challenges in disinformation detection?	-Completely agree
	-Agree
	-Neither agree nor disagree
	-Disagree
	-Completely disagree
2. Significance	
2.1 Does the question have a significant impact on evaluating disinformation/FIMI detection competencies and skills?	-Completely agree
	-Agree
	-Neither agree nor disagree
	-Disagree
	-Completely disagree
2.2 Does the question contribute to the overall goal of enhancing digital literacy?	-Completely agree
	-Agree
	-Neither agree nor disagree
	-Disagree
	-Completely disagree
2.3 Does the question play a vital role in preventing disinformation/FIMI?	-Completely agree
	-Agree
	-Neither agree nor disagree
	-Disagree
	-Completely disagree
3. Clarity	
3.1 Is the question clearly defined and easy to understand?	-Completely agree
	-Agree
	-Neither agree nor disagree
	-Disagree
	-Completely disagree
3.2 Does the question avoid technical jargon and are accessible to all users?	-Completely agree
	-Agree
	-Neither agree nor disagree
	-Disagree
	-Completely disagree
3.3 Is the question presented in a straightforward and comprehensible manner?	-Completely agree
	-Agree
	-Neither agree nor disagree
	-Disagree
	-Completely disagree

On the other hand, the results show a good level of consensus for most of the responses regarding Digcomp areas. For items 10.1, 10.2, 10.4, 10.5, the panellists agreed on a high level of knowledge among practitioners and a medium level for items 11.1, 11.2, 11.3, 11.4 related to average citizens. For items 10.3 and 11.5, there was greater dispersion in the responses. The coefficient of variation was slightly above the optimal threshold (0.5) for items 11.4 and 11.5 (Table 4). These results were complemented by the open-ended questionnaire developed for items 12 and 13. The responses to both were processed following the same procedure used in the open questionnaire of the first round (Q1).

**Table 4:** Coefficient of variation (CV) and frequency of agreement and confidence levels. Source: Own elaboration.

	fi agreement (10–7)	CV agreement	fi confident (100–75 %)	CV confident
Ítem 1 Ítem2 Ítem3 Ítem 4 Ítem 5 Ítem 6 Ítem 7 Ítem 8 Ítem 9.1 Ítem 9.2 Ítem 9.3 Ítem 9.4 Ítem 9.5 Ítem 9.6 Ítem 10.1 Ítem 10.2 Ítem 10.3 Ítem 10.4 Ítem 10.5 Ítem 11.1 Ítem 11.2 Ítem 11.3 Ítem 11.4 Ítem 11.5	100 100 100 100 100 92 85 92.3 92.3 53.8 61.5 53.8 66.7 58.3 92.3 61.5 53.8 61.5 61.5 84.6 76.9 61.5 61.5 46.1	0.18 0.19 0.15 0.17 0.19 0.37 0.31 0.30 0.13 0.38 0.45 0.47 0.41 0.50 0,12 0.19 0.24 0.27 0.26 0,33 0.35 0.44 0.53 0.53	92 92 92 85 92 92 92 85 92.3 76.9 69,2 58,3 91,7 69,2	0.16 0.13 0.15 0.20 0.21 0.12 0.23 0.14 0.17 0.19 0.32 0.26 0.23 0.22

To assess the robustness and internal consistency of expert evaluations, a Pearson correlation analysis was conducted to evaluate the relationship between the level of agreement and the level of confidence of the experts regarding the evaluated technologies. The aim of this methodological step was to test whether a higher level of agreement between the experts (measured on a Likert scale) is accompanied by a higher level of confidence in their answers (measured as a percentage) and thus supports the coherence of the experts' judgements. The analysis allowed us to determine the strength and direction of the linear relationship between these two variables. The results of the correlations (Q1: 0.68, Q2: 0.92, Q3: 0.81, Q4: 0.72, Q5: 0.84, and Q6: 0.70) demonstrate a consistently strong positive correlation across all items. This confirms that as experts' agreement with the statements increases, so does their confidence in these statements.

These findings reinforce the internal consistency of the methodological tool, demonstrating that the instrument not only gathers coherent expert input but also reflects high levels of self-reported confidence in their judgments. As such, the correlation analysis substantiates the methodological reliability of the instrument for assessing consensus on digital competence and disinformation-related curricular needs.

## Limitations

Although the number of experts participating in the Delphi panel was considerable and diverse, most of them approached the topic of disinformation from disciplinary perspectives such as communication, psychology and computer science. However, the number of experts decreased significantly when narrowing the focus to foreign information manipulation and interference (FIMI) and was even lower when considering experts with in-depth knowledge of dual-use technologies and tools that can be used to generate and disseminate malicious content.

This difficulty in finding FIMI experts with technical expertise in new technologies and dual-use technologies is a major limitation of the current study. In addition, many of the FIMI and disinformation experts showed limited awareness of the specific characteristics and future development paths of these technologies. This knowledge gap suggests that forecasting studies and technology foresight exercises need to be strengthened to support future Delphi rounds.

It is therefore recommended that this methodology be repeated in future studies with expert panels composed of specialists in emerging and dual-use technologies who have been previously trained in the dynamics and mechanisms of disinformation and FIMI. This would ensure a broader perspective that includes both the socio-communicative and technical dimensions of the problem.

## Ethics statements

This study was authorized by the Ethics Committee of Rey Juan Carlos University (Authorization ID 080720244232024) and by the Ethics Advisory Group of the EU-HYBNET project with the reference eeaG 092024. All necessary procedures for conducting the study were approved in accordance with ethical standards and the requirements established in its protocol, in relation to the research objectives. The data obtained for the analysis of the results were anonymized, ensuring the confidentiality of all participants.

## CRediT author statement

**Cristina M. Arribas:** Conceptualization, Methodology, Investigation, Data curation, Writing –original draft. **Rubén Arcos:** Conceptualization, Methodology, Supervision, Writing –review & editing, Project administration, Funding acquisition. **Manuel Gertrudix:** Conceptualization, Methodology, Supervision, Writing –review & editing, Project administration, Funding acquisition.

## Acknowledgments

This study is part of the EU-Hybnet project (Empowering a Pan European Network to counter hybrid threats) funded by the European Union's Horizon 2020 research and innovation program under agreement No883054 and has been supported by the Predoctoral Research Grant of the Rey Juan Carlos University’ own program (ID 501100007511) under the registration number PREDOC 21–008. The European Commission’s endorsement of this publication does not constitute an approval of its contents, which reflects the views only of the authors, and the Commission cannot be held responsible for any use which may be made of the information contained therein.

## Declaration of competing interest

The authors declare that they have no known competing financial interests or personal relationships that could have appeared to influence the work reported in this paper.

## Data Availability

All the data are available on Zenodo repository. All links for the data are included in the manuscript.
